# Black Tea Extracts/Polyvinyl Alcohol Active Nanofibers Electrospun Mats with Sustained Release of Polyphenols for Food Packaging Applications

**DOI:** 10.3390/polym15051311

**Published:** 2023-03-06

**Authors:** Lucía M. Quintero-Borregales, Alicia Vergara-Rubio, Ayelen Santos, Lucía Famá, Silvia Goyanes

**Affiliations:** 1 Departamento de Física, Facultad de Ciencias Exactas y Naturales, Universidad de Buenos Aires, Buenos Aires C1428EGA, Argentina; 2 Instituto de Física de Buenos Aires (IFIBA), CONICET—Universidad de Buenos Aires, Buenos Aires C1428EGA, Argentina; 3 III-UNSAM-CONICET, Instituto de Investigación e Ingeniería Ambiental, Escuela de Hábitat y Sostenibilidad, Campus Miguelete, 25 de Mayo y Francia–San Martín, Buenos Aires B1650CBG, Argentina

**Keywords:** polyvinyl alcohol mats, black tea extracts, electrospinning, polyphenols encapsulation, polyphenols extended-release, antioxidant activity, food active packaging

## Abstract

The efficiency in the capabilities to store and release antioxidants depends on the film morphology and its manufacturing process, as well as on the type and methodology used to obtain the polyphenol extracts. Here, hydroalcoholic extracts of black tea polyphenols (BT) were obtained and dropped onto different polyvinyl alcohol (PVA) aqueous solutions (water or BT aqueous extract with and without citric acid, CA) to obtain three unusual PVA electrospun mats containing polyphenol nanoparticles within their nanofibers. It was shown that the mat obtained through the nanoparticles precipitated in BT aqueous extract PVA solution presented the highest total polyphenol content and antioxidant activity, and that the addition of CA as an esterifier or PVA crosslinker interfered with the polyphenols. The release kinetics in different food simulants (hydrophilic, lipophilic and acidic) were fitted using Fick’s diffusion law and Peppas’ and Weibull’s models, showing that polymer chain relaxation is the main mechanism in all food simulants except for the acidic, which presented an abrupt release by Fick’s diffusion mechanism of about 60% before being controlled. This research provides a strategy for the development of promising controlled-release materials for active food packaging, mainly for hydrophilic and acidic food products.

## 1. Introduction

The circular economy is the upcoming trend in the packaging industry as a result of the accumulation of plastic waste and pollutants that produce catastrophic results for the environment [1,2]. International organizations recognize plastic marine pollution as an obstacle to sustainable development. Companies seeking to be part of the sustainable development goals (SDGs) have proposed the use of biodegradable components to replace or reduce the use of traditional single-use plastics [3]. In addition, market surveys have shown consumer desire for sustainable materials that also have functional properties [4]. In this context, the electrospinning technique emerges as a very attractive alternative to obtain active ultralight packaging. Electrospun mats consist of entanglements of nanofibers with void spaces between them (forming a non-woven fabric) that can encapsulate different active components inside or on the surface of the fibers, and/or in the space between them [5]. It was demonstrated that the encapsulation of polyphenols led to higher microbiological stability and better control of their release from the mat [6,7,8,9]. The use of nanostructured mats with encapsulated polyphenols as biodegradable packaging is highly desirable to extend the shelf life of food [10]. As the electrospinning technique involves the processing of a polymer solution or dispersion at room temperature, it allows for the incorporation of thermolabile active components obtained from natural sources, in line with new ecological trends [11,12,13].

Polyvinyl alcohol (PVA) is the most widely used polymer in electrospun mats because it is biodegradable, inexpensive, Generally Recognized As Safe (GRAS), and easily electrospinnable. Furthermore, PVA has the ability to encapsulate different antioxidant agents from natural extracts [14,15,16,17]. As PVA is water soluble, its electrospinning process is environmentally friendly. Different treatments have been reported in the literature to increase the stability in water of PVA mats [18], including the use of temperature [19,20] or crosslinking/esterifying the mats with citric acid [21,22]. Both methods led to insoluble materials that remained hydrophilic without damaging the encapsulated actives [21,23]. Estevez-Areco et al. (2018) demonstrated that the antioxidant properties of PVA mats with encapsulated rosemary polyphenols remained intact after insolubilization. These mats also exhibited a high release of polyphenols in different food simulants [15]. Zeinali et al. (2021) encapsulated jujube extract in a PVA electrospun mat and showed a great effect in the preservation of strawberries [9]. Most investigations prepare active mats by incorporating aqueous polyphenolic extracts into the electrospinning solution [10]. The incorporation of the ethanolic extract of polyphenols is also possible by dripping it onto the aqueous polymer solution (solvent displacement technique). This technique leads to obtaining polyphenol nanoparticles in the polymer fibers of the mats. Estevez-Areco et al. (2018) applied this idea by dripping an ethanolic rosemary extract in an aqueous PVA solution and observed that the mat containing the polyphenol nanoparticles presented a higher antioxidant activity and had greater control in the polyphenols’ release [15].

The antioxidant power of black tea polyphenols from the Camellia sinensis plant has attracted attention because they can inhibit food’s oxidation by delaying microbial proliferation [24]. Different researchers reported that the antioxidant activity of green tea is greater than black tea; possibly, for this reason, the literature focuses mainly on the use of green tea extracts in different polymers [25,26]. Examples of this are the works of Lan et al. (2022), who used different concentrations of green tea extract in a gelatin mat, and Luo et al. (2020), who employed it in PVA nanofiber mats [27,28]. Black tea differs from green tea in the class of polyphenols that can be obtained. In green tea, the dominant compound is catechin ((-)-epigallocatechin-3-gallate), whereas flavins are the main polyphenol in black tea [29]. Black tea is the most consumed tea worldwide (78%); it is usually prepared by fermenting the crushed withered leaves. In this process, oligomers known as theaflavins and large polymeric compounds such as thearubigins are produced [29,30]. Black tea has lower amounts of monomeric polyphenols and higher concentrations of oligomers and polymeric compounds with lower volatility than green tea. Thus, it is likely that it will better support any process to incorporate it into a polymeric matrix, particularly electrospinning. Despite this, few studies propose the use of black tea extracts [31]. Rajapaksha et al. (2021) incorporated an ethanol:water extract of spent black tea into a starch matrix, showing that the microencapsulation of the polyphenols protects the antioxidant compounds during the film processing and produces a significant migration of the active compounds into both aqueous and fatty food simulants [32]. Moreover, Ashrafi et al. (2018) obtained chitosan active films with 1–3 wt% of black tea (kombucha tea) in an aqueous extract, enhancing significantly the antioxidant activity (59%) for the highest concentration of tea and significantly retarding microbial growth, extending the food shelf life [33]. Nevertheless, polymer systems with black tea polyphenols have never been processed by electrospinning. In addition, very few works have incorporated polyphenols in the form of nanoparticles through an in situ synthesis in the electrospinning polymeric aqueous medium [15,34]. To date, no research has employed the formation of polyphenolic nanoparticles by the solvent displacement method in a PVA solution as a strategy to maximize the amount of polyphenols where the solvent is an aqueous extract from the same source of polyphenols as the nanoparticles. Unbelievably, to date black tea has not yet been considered in such a matter.

Here, we hypothesize that using the black tea extract’s polyphenol nanoparticles formed in situ in an aqueous PVA solution, where the solvent is black tea extract, can maximize the total polyphenol content and antioxidant activity of an electrospun mat. In addition, we exhibited that heat treatment can be performed to achieve water insoluble PVA mats, avoiding the use of citric acid as esterifier or crosslinker. The presented results are significant because they demonstrate that electrospun mats obtained with nanoparticles precipitated on a black tea aqueous extract PVA doubling the total extracted polyphenols content with about 24% higher antioxidant activity than those containing nanoparticles formed in a water-PVA solution. Moreover, the release in both mats exceeded 64% in the hydrophilic simulant and 98% in the lipophilic one. In the case of the mat with citric acid, there could be an interaction with the polyphenols, inhibiting their complete release. The polyphenols’ kinetic release of the mats in food simulants (FS, hydrophilic, lipophilic and acidic) was fitted by Fick’s diffusion law and Peppas’ and Weibull’s models, showing that polymer chain relaxation is the leading mechanism in all FS, except for the mat with CA that presented an abrupt release by Fick’s diffusion mechanism in the acid medium. 

This study is original because it proposes a new strategy for the development of active nanofibrous PVA mats, which could be very promising as a type of packaging to extend the shelf life of food products.

## 2. Materials and Methods

### 2.1. Materials

Black tea (Green hills) was purchased at a local market in Buenos Aires, Argentina; polyvinyl alcohol (Mowiol 10–98, Mw = 61,000), 1,1-diphenyl-2-picrylhydrazyl (DPPH∙) and 6-hydroxy-2,5,7,8-tetramethylchroman-2-C (TROLOX) were obtained from Sigma-Aldrich (St. Louis, MO, USA). The Folin-Ciocalteu reagent and anhydrous p.a. gallic acid were purchased from Anedra and Biopack in Buenos Aires, Argentina, respectively, and ethanol 96% *v*/*v* was bought from Droquimar (Buenos Aires, Argentina).

### 2.2. Black Tea Extracts

#### 2.2.1. Black Tea Aqueous Extract

The black tea aqueous extract (BT_Aq_) was obtained following the procedure mentioned by Bruni et al. (2020) with some modifications; 30 g of black tea was dispersed in 100 mL of distilled water at 100 °C for 40 min [35]. The aqueous extract was cooled to room temperature and filtered (mat pore size 0.8 μm). The remaining solution was newly filtered through a 0.45 μm pore nylon membrane (MSI, WESTBORO, Massachusetts, USA). The solution was kept in amber sealed containers and refrigerated until further use.

#### 2.2.2. Black Tea Ethanolic Extract

The polyphenols’ ethanolic extract from black tea (BT) was obtained following the method described by Estevez-Areco et al. (2020) with some modifications, 10 g of black tea were dispersed in 50 mL of a mixture of ethanol:water (70:30) at 50 °C for 55 min. The ethanolic extract was then cooled and filtered following Section 2.2.1 [36]. 

#### 2.2.3. Solid’s Extraction Yields

The solid’s extraction yield was calculated gravimetrically following the method used by Estevez-Areco et al. (2018): 5 mL of each extract was dried at 50 °C until constant weight [15]. 

#### 2.2.4. Total Polyphenol Content (TPC) and Antioxidant Activity

The total polyphenol content (TPC) of all extracts was determined by the Folin-Ciocalteu method employing the UV-Vis technique at 760 nm (spectrophotometer UV-1800 SHIMADZU, Tokyo, Japan). Absorbance results were compared to a calibration curve built using gallic acid as a standard (10–150 mg/L) and the TPC was reported as gallic acid equivalents (GAE/mL of each extract). 

Antioxidant activity of every sample was obtained following the method proposed by Brand-Williams et al. (1995) and using Equation (1) [37].

(1) $$DPPH\;inhibition\;(\%)=100\frac{A_{b}-A_{s}}{A_{b}}$$

where A_b_ is the absorbance of DPPH at the initial time and A_s_ is the absorbance of DPPH∙at 30 min. 

The absorbance results were compared to a calibration curve built with TROLOX (in ethanol) as a free radical reagent (50–350 mg/L). Furthermore, the concentration of polyphenols needed to decrease DPPH∙concentration by 50%, also known as efficient concentration (EC50), was obtained, and both results were expressed in μmol TROLOX equivalent (μg TE/mL of extract).

The solid’s extraction yield, total polyphenols concentration (TPC), antioxidant activity and EC50 of both extracts are shown in Table 1.

As can be seen in Table 1, both the solid’s yield and the antioxidant activity are much higher for BT than for BT_Aq_. BT doubled the solid’s extraction yield and increased the antioxidant activity by one order. This agrees with the literature, which has shown that with ethanol:water mixtures, the extraction of polyphenols is enhanced [38,39]. Possibly this is a consequence that the alcohol-soluble polyphenols, as well as the water-soluble polyphenols, are extracted.

### 2.3. Preparation of Nanoparticles and Electrospun Mats

Four electrospinning PVA systems were prepared. One of them consisted of PVA in water and another three containing black tea polyphenols’ nanoparticles in different aqueous PVA solutions (water or BT_Aq_, with and without citric acid). PVA solutions were first heated at 80 °C for 1 h under constant stirring and then allowed to cool to room temperature. For the case of the nanoparticle systems, the nanoparticles were formed in situ in the different PVA solutions using the solvent displacement technique. For this, a black tea ethanolic extract (BT) was incorporated dropwise to the aqueous PVA solution (named: PVA + BT_NP_) and the aqueous black tea extract PVA solution, with or without 5 wt% (5 g/100 g of PVA) of citric acid (CA) (PVA + CA + BT_Aq_ + BT_NP_ and PVA + BT_Aq_ + BT_NP_, respectively). As expected, these solutions become turbid as a consequence of the nanoparticles’ precipitation due to the solvent displacement phenomenon. 

The composition of all the PVA solutions to be electrospun, as well as their electrical conductivity and viscosity, measured at room temperature using an Orion™ Versa Star Pro™ (Thermo Fisher Scientific, Waltham, MA, USA) and a Brookfield viscometer model LV DV-E™ (AMETEK Brookfield, Marlborough, MA, USA), respectively, are shown in Table 2.

PVA mats were obtained using an in-house electrospinning equipment [40]. The solutions were placed in a 10 mL plastic syringe connected to a six-needle injector with a 0.8 mm inner diameter. They were then ejected at a rotating drum collector (6 cm in diameter) and placed at 12.5 cm from the needles by applying a 28 kV between the collector and the needles. The syringe was placed at a syringe pump PC11U (APEMA, Buenos Aires, Argentina) with a controlled feed of 2.5 mL/h. Once the mats were obtained, a thermal treatment (190 °C for 10 min) was performed to improve their solubility in water [22,40,41].

**Figure 1:**
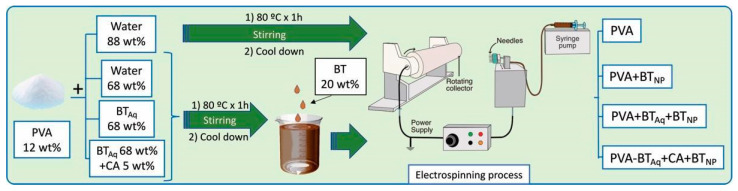
Scheme of the preparation of solutions and electrospinning process.

A schematic of the preparation of the electrospinning solutions and active PVA electrospun mats is shown in Figure 1.

### 2.4. Characterizations of Nanoparticles and Mats

#### 2.4.1. Nanoparticles Size and Mats Morphology

The size of the BT nanoparticles and fiber’s diameter were analyzed by field emission scanning electron microscopy (FEG-SEM, FEI-Quanta^TM^ 250 FEG, Hillsboro, OR, USA). Nanoparticle samples were obtained by dripping ethanolic extract on water and on BT_Aq_. Drops of these dispersions were placed on single crystal silicon pieces which were dried at 50 °C for 24 h and sputter-coated with platinum using an Edward S150B sputter coater, Heathfield, UK (70 s, 0.06 mbar of Ar); mats samples were placed in carbon tapes and sputter-coated with platinum (40 s, 0.06 mbar of Ar) before SEM observations. Finally, the nanoparticles’ size and fiber’s diameter, which was considered cylindrical [15], were determined by measuring the diameter of at least 100 nanoparticles and 200 fibers from the SEM images, using ImageJ (free software by Wayne Rasband, National Institutes of Health, NIH in USA).

#### 2.4.2. FTIR

Absorbance spectra were obtained using an Attenuated total Fourier Transform Infrared (ATR-FTIR 4100 spectrophotometer, Jasco Inc., Hachioji, Japan), in the range of 4000 to 650 cm^−1^ at a resolution of 4 cm^−1^ and the average of 64 scans. All mats’ spectra were normalized using the C-H band (1372 cm^−1^), which corresponds to a functional group that is not expected to interact with any of the components [15]. For comparative purposes, powdered extracts samples were tested and their spectra were normalized using the 844 cm^−1^ band as it does not change. Tests were carried out in triplicate with no significant differences.

#### 2.4.3. Contact Angle (θ)

Essays were carried out to study the hydrophobicity of the mats [42]. For the essays, a “one attention theta lite optical tensiometer” (Biolin Scientific, Västra Frölunda, Sweden) was used at room temperature, dripping a drop of water (~13 μL) on the surface of each material. The contact angle (θ) was determined as the angle formed by the intersection of the drop of water-surface of the mat and the liquid-steam (tangent on the boundary of the drop). The mean of the test was reported with five measurements.

#### 2.4.4. Water Solubility (S)

The water solubility test was performed by taking ~25 mg (*m_si_*) of each mat and submerging it in 50 mL of distilled water at room temperature for 24 h under constant stirring; then was dried at 100 °C for 24 h and weighed (*m_sf_*). Results were calculated following Equation (2) and expressed as the average of three samples.

(2) $$S=\frac{m_{si}-m_{sf}}{m_{si}}$$

#### 2.4.5. Total Extracted Polyphenol Content (TEPC) and Antioxidant Activity of Mats

To measure the TEPC in the mat and antioxidant activity, a polyphenol extraction from all mats was performed. For TEPC, a square of 25 mg of each mat was placed in 100 mL of ethanol:water solution (70:30 *v*/*v*) at 50 °C with constant stirring and reflux for 32-h. The polyphenol content and antioxidant activity were measured by the Folin-Ciocalteu and DPPH∙methods, respectively. Briefly, 0.4 mL of each sample was mixed with 2 mL of Folin-Ciocalteu reagent (previously diluted 1:10), and 1.6 mL of sodium carbonate (7 g/100 mL) was incorporated into the mix afterwards. Every mixture was allowed to proceed for 30 min in the darkness. Then, TEPC and the antioxidant activity were determined following the procedure described in Section 2.2.4.

Theoretical TPC, also known as the total polyphenol content loaded (TPL), for each system was calculated for comparative purposes, obtaining ~26.9 mg GAE/PVA + BT_NP_ mat and ~47.6 mg GAE/PVA + BT_Aq_ + BT_NP_ mat (with and without citric acid, respectively) in 100 mL of the precursor solution. 

To evaluate the stability of the extracts released from the PVA mats, TEPC and antioxidant activity were determined after 4 months of storage at room temperature.

#### 2.4.6. Release of Polyphenols from the Mats

Polyphenols release from the mats was studied in three different food simulants: hydrophilic (ethanol 10% *v*/*v*), lipophilic (ethanol 50% *v*/*v*) and acidic (acetic acid at 3% *v*/*v*) [43]. Samples of ~25 mg of each mat were immersed in 20 mL of each food simulant and shaken at 120 rpm. Aliquots were taken at different times (20 s, 20 min, 40 min, 1 h, 2 h, 4 h, 6 h, 24 h, 48 h and 72 h) and polyphenols released were measured using the Folin-Ciocalteu method described in Section 2.4.5. Furthermore, the released polyphenols (%) were calculated using Equation (3), where *M_pt_* is the amount of the polyphenol released at time *t* and *M_o_* is the total extracted polyphenol content (TEPC) normalized to the mass of the mat. The results are reported as the mean of three essays and then fitted with Fick’s diffusion law and the Weibull function using SciPy [15].

(3) $$Released\;polyphenols\left(\%\right)=\frac{M_{pt}}{M_{o}}\;100$$

If the system is an electrospun mat, nanofibers must be considered as cylindrical geometry and the Fick’s equation to the second law of diffusion solution (Equation (4)) is [44]:

(4) $$\frac{M_{t}}{M_{\infty }}=1-\sum_{i=1}^{\infty }\frac{4}{r^{2}\alpha_{n}^{2}}exp\left(-D\;\alpha_{n}^{2}\;t\right)$$

Here *M_t_* is the relation, *M_pt_*/*M_o_*, *M_∞_* is the polyphenol amount at equilibrium (infinite time), *D* is the diffusion coefficient, *r* is the fiber average radius and *r⋅α_n_* are the Bessel’s positive roots to this function of first kind of zero order [15]. In this work the first ten roots of the Bessel function were significant for the fitting. For systems that do not follow a pure diffusional process, the Power Law [45] by Korsmeyer-Peppas is an alternative. This law only considers the first portion of the release where *M_t_*/*M_∞_* < 0.6 and can describe the Fickian and non-Fickian release based on the parameters of Equation (5):

(5) $$\frac{M_{t}}{M_{\infty }}=k\;t^{n}$$

Here *k* is the kinetic constant and *n* a diffusional exponent, which characterizes the release system mechanism. If *n* is lower than 0.5 it represents a pseudo-Fickian diffusion mechanism, close to 0.5 the principal mechanism is Fick diffusion, close to 1 it indicates Case II transport (where polymer chain relaxation domains polyphenols release), and when *n* is between 0.5 and 1 it denotes an anomalous release. Another alternative to describe the release behavior is the empirical Weibull function (Equation (6)):

(6) $$\frac{M_{t}}{M_{\infty }}=1-exp\;(a\;t^{b})$$

Here *a* and *b* are constants. A *b* value smaller than 0.75 indicates that the release mechanism occurs according to Fick’s diffusion Law combined with Case II transport, intermediate values (0.75–1) indicate Case II transport and *b* > 1 indicate complex release mechanisms [46,47].

### 2.5. Data Treatment and Statistical Analysis

All obtained results were analyzed through two-way ANOVA with a 95% confidence level (*p* < 0.05) and Tukey’s Post-Hoc test. Data were reported as the main and standard deviation.

## 3. Results and Discussion

### 3.1. Characterizations of Nanoparticles and Mats

#### Nanoparticles Size and Mats Morphology

**Figure 2:**
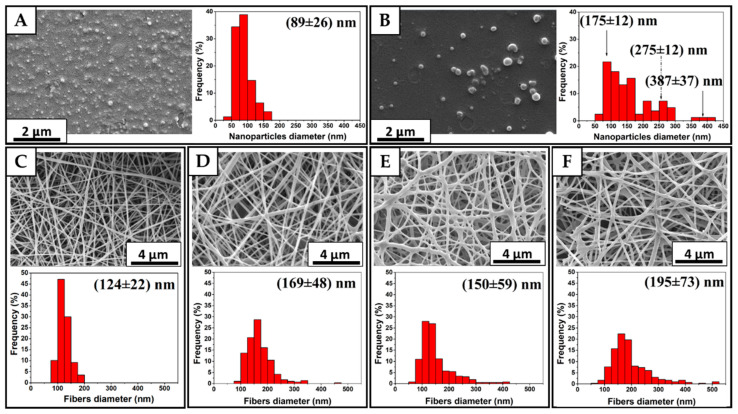
SEM images and histograms diameters of nanoparticles (**A**) BT_NP_ and (**B**) BT_Aq_ + BT_NP_, and mats’ surface (**C**) PVA, (**D**) PVA + BT_NP_, (**E**) PVA + BT_Aq_ + BT_NP_ and (**F**) PVA + BT_Aq_ + CA + BT_NP_.

Figure 2 shows the micrographs of the nanoparticles produced by dripping the ethanolic extract on an aqueous solution of PVA (Figure 2A) and on BT_Aq_ (Figure 2B). They reveal the enormous effect of the type of non-solvent used on the sizes of nanoparticles. While the first one exhibits one population with a diameter of 89 ± 26 nm, the other one shows a wider size distribution with three populations: one with smaller particles (around 175 nm), the second with intermediate sizes (around 275 nm), and the third with nanoparticles with an average size of 387 nm. López-Cordoba et al. (2017) reported similar results when different non-solvents were involved [34]. The use of CA did not present significant differences in the nanoparticle size distribution with respect to BT_Aq_ + BT_NP_ (Figure 2B). 

All mats exhibited a homogeneous fibers morphology, as shown in Figure 2C–F. However, it can be noted that the main diameter of the nanofibers and their size distribution depend on the solution studied. It is known that the evaporation of the solvent in the polymeric solution plays a fundamental role in the final morphology of the electrospun mats. According to the literature, a faster solvent evaporation rate leads to wider nanofiber diameters, regardless of the polymer used in the electrospinning process [48,49,50]. This effect is associated with the earlier solidification of the nanofiber that is expelled from the needle: the higher the evaporation rates, the lower the elongation of the nanofiber, solidifying before others that have had more time to stretch (resulting in a narrower nanofiber). PVA + BT_NP_, which has water, polyphenols and ethanol, contrary to the PVA mat that only contains water, showed an increase in the average fiber size (Figure 2C). Estevez-Areco et al. (2018) reported that the addition of ethanol as a solvent in the electrospinning solution decreases the surface tension of the PVA solutions and consequently the evaporation rate during the electrospinning process increases, leading to wider nanofiber diameter and size distributions [15].

The mean diameter of the PVA + BT_Aq_ + BT_NP_ nanofiber (Figure 2E) is greater than that of PVA but slightly lower than that of PVA + BT_NP_. This material contains the greatest amount of polyphenols but has the highest electrical conductivity (Table 2), which, according to the literature, leads to thinner fibers [5]. Moreover, in PVA + BT_Aq_ + BT_NP,_ several fibers were stuck together. However, when citric acid was present in the same solution (Figure 2F), the diameter of the fiber increased to 195 ± 73 nm, leading to the highest value among the active PVA mats. As was reported, when CA concentration increases in an electrospinning precursor solution, the conductivity decreases, leading to a notably lower nanofiber diameter and a higher dispersion [51,52]. This effect has been associated with the polyphenols avoiding the PVA chains from interacting via inter/intramolecular hydrogen bonds and creating void spaces with the polyphenols entrapped within, leading to wider fibers [27,41,49,50]. The wider dispersion of the fibers’ diameter obtained for the mats prepared with both extracts (PVA + BT_Aq_ + BT_NP_ and PVA + BT_Aq_ + CA + BT_NP_) was possibly due to the presence of water and ethanol soluble polyphenols, contrary to PVA + BT_NP_ in which only water soluble polyphenols are present. 

### 3.2. FTIR

**Figure 3:**
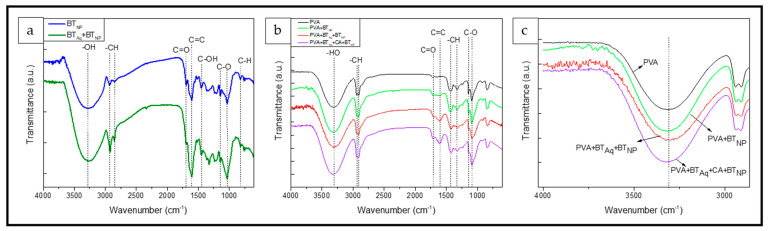
FT-IR of (**a**) the powder extracts, (**b**) all mats obtained and (**c**) detailed differences between PVA and PVA with polyphenols mats between 4000 and 650 cm^−1^.

Figure 3a shows FTIR spectra of BT_NP_ and BT_Aq_ + BT_NP_ powder and the characteristic polyphenol bands. The wide bands around 3282 cm^−1^, 1142 cm^−1^ and 1032 cm^−1^ are attributed to the stretching of -OH and C-O groups and the bending of the C-OH bonds, respectively, from phenol groups present in the extracts [18,28,53]. The bands at 2922 and 2848 cm^−1^ are ascribed to symmetric and asymmetric C-H stretching, and the band at 823 cm^−1^ corresponds to the C–H deformation out of the plane. The bands around 1695 cm^−1^ and 1607 cm^−1^ are associated to C=O from carbonyl groups and C=C stretching vibration of the aromatic rings, respectively. Finally, the band around 1448 cm^−1^ can be assigned to the vibrations of aliphatic hydroxyl groups [54]. As expected, all the bands associated with the polyphenols show a greater increase in their intensity in BT_Aq_ + BT_NP_ due to their higher TPC (Table 1). The intensity of some bands differs depending on the extract, suggesting that they have different types of polyphenols, which is expected due to the different extraction methods used. In this sense, the presence of new bands at 1327 and 1367 cm^−1^ is observed in the BT_Aq_ + BT_NP_ that have been associated with the C-O interactions and the O-H stretching of the polyphenols, respectively [55].

Several changes have been produced by the incorporation of the polyphenols into the PVA mats (Figure 3b). Neat PVA mat spectrum showed a band around 3304 cm^−1^ corresponding to the stretching of the hydroxyl groups. Furthermore, there is the double peak in 2938 cm^−1^ and 2908 cm^−1^ that can be assigned to the symmetric and asymmetric C-H stretching. The band around 1707 cm^−1^ corresponds to C=O, 1088 cm^−1^ to the stretching of the O-H groups and that at 1328 and 1431 cm^−1^ to the C-H bending and that around 1142 cm^−1^ to the C-O bending [41,56]. The black tea extract caused increases in the O-H absorption band of PVA mats and the appearance of a new band around 1606 cm^−1^ associated with C=C vibrations of the aromatic ring of the extract, confirming the presence of polyphenol compounds [28,56]. On the other hand, a broadening in the band at 1088 cm^−1^ for mats containing the black tea extract was observed (Figure 3b), possibly due to an overlap with the C-OH band from phenolic compounds [15]. 

Different effects can be observed in the range of the OH vibration (Figure 3c): almost no displacement in the case of the PVA + BT_NP_ mat, which could be due to the lower polyphenol content, and a shift to lower wavenumber in the PVA + BT_Aq_ + BT_NP_ mat that could be due to hydrogen bonding interactions between PVA and black tea extract components [28]. However, in the PVA + BT_Aq_ + CA + BT_NP_ mat, this shift is reverted possibly due to the esterification/crosslink reaction between CA and -OH groups of the PVA chain [15,41,57]. This effect is also observed by the increase in the intensity of the C=O stretching band. The intensity ratio of the C=O band to the C-H one (I_1707_/I_1374_) for PVA + BT_Aq_ + CA + BT_NP_ (6.96) is higher than that of the PVA + BT_Aq_ + BT_NP_ (5.03), PVA-BT_NP_ (4.42) and PVA mat (2.06) samples. On the other hand, polyphenols can also interact with the -OH groups in the PVA mat, as shown above. These interactions may help to retain the polyphenols within the fibers, as was also observed for PVA with the CA mat [15]. These results, as will be shown in Section 3.5, correlated with the lower content of polyphenols extracted from this mat.

### 3.3. Contact Angle (θ)

**Figure 4:**
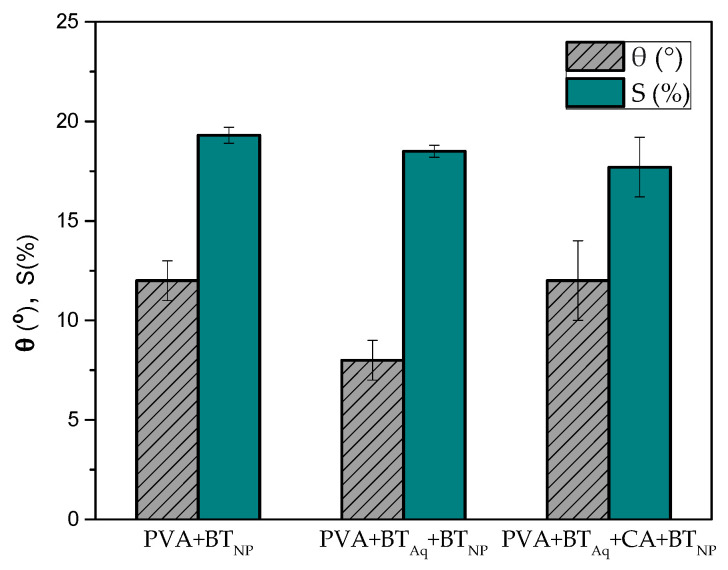
Contact angle (θ) and water solubility (S) for the developed mats.

Contact angle (θ) measurements are reported in Figure 4. The PVA + BT_NP_ mat shows lower θ than the PVA mat, indicating an increase in its hydrophilicity, possibly due to the nanoprecipitation that leads to a higher surface-volume ratio of the BT nanoparticles [34].

On the other hand, PVA + BT_Aq_ + BT_NP_ had the lowest θ value; this can be associated with the incorporation of the aqueous polyphenol BT, suggesting that more available OH are on the surface. Moreover, the addition of CA could lead to an esterification/crosslink reaction with PVA, increasing the PVA + BT_Aq_ + CA + BT_NP_ mat contact angle with respect to PVA + BT_Aq_ + BT_NP_, and thus, its hydrophilicity [15,23,41].

### 3.4. Water Solubility (S)

Water solubility (S) increases with the incorporation of the polyphenols (Figure 4). Results show a slight difference between PVA + BT_NP_ and PVA + BT_Aq_ + BT_NP_ but do not show significant differences between the mats with and without citric acid. This agrees with Liu et al. (2019), who attributed this effect to polyphenols content in the electrospun mat [58].

### 3.5. Total Polyphenol Extracted Content (TEPC) and Antioxidant Activity of Mats

Comparing the samples of the PVA + BT_NP_ and PVA + BT_Aq_ + BT_NP_ mats, the first has the lowest TEPC (Table 3). This was expected since the only source of polyphenols is BT. However, considering the theoretical TPC (Section 2.4.5) used in the precursor solutions, 88.9 ± 0.4% and 88.2 ± 0.3% of polyphenols’ retention, respectively, were achieved. The difference between the theoretical data for these mats could be due to the loss of polyphenols during the electrospinning process and/or the heat treatment. Citric acid (PVA + BT_Aq_ + CA + BT_NP_) hindered the extraction of the polyphenols from the mat, releasing only 31.0 ± 0.4%. This effect may have been due to possible interactions between citric acid and polyphenols. Since CA interacts with PVA [15,17], the polyphenols could have been retained in the mat.

Moreover, Table 3 shows higher antioxidant activity for PVA + BT_Aq_ + BT_NP_ followed by PVA + BT_NP_, which presented similar values to that reported by Estevez-Areco et al. (2018) for PVA mats with polyphenols from another natural source (120 ± 8 μmol/g of mat) [15]. In both cases, the polyphenol nanoparticles were precipitated over an aqueous PVA electrospinning solution. However, the results obtained for PVA + BT_Aq_ + BT_NP_ mat showed that when the nanoparticles were precipitated on an aqueous black tea extract PVA solution, the TEPC and the antioxidant activity were increased. 

The stability of the mats with higher total extracted polyphenols content (PVA + BT_NP_ and PVA + BT_Aq_ + BT_NP_) after 4 months of storage was evaluated. As can be seen in Table 3, the TEPC and the antioxidant activity of these mats slightly decreased (less than 1%). These results suggest that the polyphenols remained stable for at least during the studied storage time.

### 3.6. Release of Polyphenols from the Mats

The release test provides information on the affinity between active materials and food products; thus, the most suitable active material can be selected for each type of food [32,59]. The compositions of polymeric composites and the food simulant determine the migration mechanisms between both phases. The chemical potential of each phase controls the diffusion coefficients and the equilibrium concentrations.

**Figure 5:**
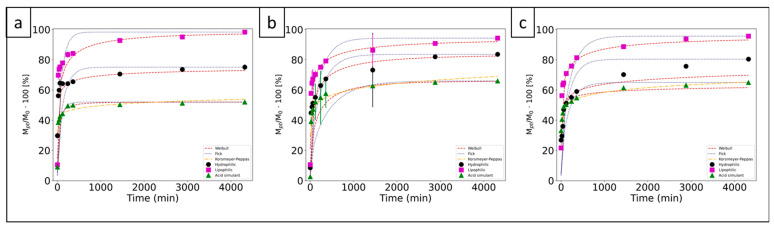
Polyphenols release of (**a**) PVA + BT_NP_, (**b**) PVA + BT_Aq_ + BT_NP_, and (**c**) PVA + BT_Aq_ + CA + BT_NP_ mats normalized at the amount of the releasable polyphenol *M_0_* and fitted by applying Fick’s diffusion law and the Weibull model, and Peppas’ model on the release kinetics curves where it applied.

The release of polyphenols, normalized with respect to the TEPC, in the hydrophilic simulant was from 75% to 84%, in the lipophilic from 94% to 98% and in the acid from 52% to 66% (Figure 5). The lipophilic medium showed the smallest difference in the final release. 

The results of the polyphenols released in percent based on the TPL and the TEPC are reported in Table 4. As was expected, the polyphenols release based on TPL from PVA + BT_NP_ and PVA + BT_Aq_ + BT_NP_ was lower than that based on TEPC for all food simulants. When CA was present in the mat (PVA + BT_Aq_ + CA + BT_NP_), the polyphenols release based on TPL tripled. This effect is due to the esterification/crosslinking reaction between CA and the -OH groups of the PVA chain as previously discussed.

For all mats, the highest release occurred in the lipophilic simulant. Black tea nanoparticles were prepared in ethanol:water (70:30); therefore, they contain a large amount of ethanol-soluble polyphenols that can be released in a lipophilic simulant [17]. 

On the contrary, all mats showed a lower and more controlled polyphenol release in hydrophilic and acidic simulants (reaching its maximum at 72 h), indicating that they could be used as packaging for hydrophilic and acidic foods, providing them with a controlled release of polyphenols for a long time. The lowest release was observed in the acid simulant. This has been associated with the insolubility of nanoparticles in these simulants [15]. As it has been shown in Table 1, the TEPC and the antioxidant activity from PVA + BT_Aq_ + BT_NP_ are lower than PVA + BT_NP_ and, comparing Figure 5a,b, higher release values between them are observed in the hydrophilic simulant. It might be related to the polyphenols’ presence in the aqueous phase since they were more hydrophilic than those made from the ethanolic extract. 

When citric acid is used (PVA + BT_Aq_ + CA + BT_NP_), the amount of released polyphenols was highest at 72 h in the lipophilic simulant, resulting in around 98% of the TEPC of this mat (being TEPC 31%, as discussed in Section 3.5, Figure 5c). This suggests that polyphenols in this mat are more soluble in a nonpolar simulant. In the hydrophilic simulant, the release is lower than the PVA + BT_Aq_ + BT_NP_ one. This could be related to a higher chemical affinity between the simulant and the polyphenols with the citric acid presence. 

Furthermore, in the acid simulant, a faster release of polyphenols was observed (Figure 5c). As previously discussed, CA can interact with both polyphenols and PVA, interrupting some interactions between polyphenols and PVA, thus, leaving free polyphenols to be more easily released. 

Fick’s diffusion law and Weibull’s model were applied on all the release kinetics curves, and the Power Law model was only applied to the polyphenols release curves that met the method’s condition (acid simulant). This method did not correctly fit the curves for the hydrophilic and lipophilic simulants as only five or fewer spots achieved release values less than 60%. The results (Table 5) suggested that Weibull’s model is the appropriate one for all mats and Fick’s diffusion model was only proper for the PVA + BT_Aq_ + CA + BT_NP_ mat in an acidic simulant. This could be explained by the close affinity between the simulant and the acidic mat. The value of the diffusion coefficient D = 3.3 ± 0.7 × 10^−15^ cm^2^/s was higher than in the previous works with rosemary polyphenols extract [15], indicating a faster releasing process. Analyzing the b values of Weibull’s model, all the mats’ releases present a combined mechanism of a Fickian diffusion with a Case II transport. These results could indicate that polymer chain relaxation by the Case II transport is the principal mechanism in the polyphenol release since Fick’s model was not adequate except for PVA + BT_Aq_ + CA + BT_NP_ in the acid medium. In this case, the Fickian diffusion mechanism in the combination could be the main one. 

## 4. Conclusions

The investigation of ultralight and environmentally friendly active materials obtained through an emerging technique, such as electrospinning, is a very attractive alternative to replace petrochemical plastics in the packaging industry. In the present study, antioxidant electrospun mats were obtained by the solvent displacement technique, dripping ethanolic black tea extract onto three PVA solutions (water or aqueous extract of black tea, with and without CA). Our work demonstrated that the best strategy to maximize the total polyphenol content and the antioxidant activity of electrospun mats was to form black tea polyphenolic nanoparticles in a PVA-black tea aqueous extract solution (PVA + BT_Aq_ + BT_NP_), due to the synergy between the polyphenols of the nanoparticles and those from the PVA solution.

Regardless of the techniques developed in this work for the incorporation of black tea antioxidants in electrospun mats, all materials achieved the most controlled polyphenols release in hydrophilic and acid simulants. By applying Fick’s diffusion law, and Peppas’ and Weibull’s models, on the release kinetics curves, it was shown that polymer chain relaxation was the principal mechanism in black tea polyphenol release.

This investigation provides an original and attractive strategy to achieve highly promising ultralight materials with controlled-released polyphenols for use as active and environmentally friendly food packaging.

## Figures and Tables

**Table 1:** Characterization of the obtained extracts.

Extract	BT_Aq_	BT
Solid’s extraction yield (g of solids/L of extract)	51.2 ± 0.9 ^a^	102 ± 1 ^b^
TPC (mgGAE/mL of extract)	4.0 ± 0.1 ^a^	17.6 ± 0.2 ^b^
TPC (mgGAE/100 g of electrospun solution)	272 ± 10 ^a^	352 ± 20 ^b^
Antioxidant activity (μmol/mL)	16 ± 4 ^a^	124 ± 4 ^b^
EC50 (μg/mL)	111 ± 6 ^a^	80 ± 4 ^b^

BT_Aq_: Black tea aqueous extract; BT: Black tea ethanolic extract. ^a,b^ Different letters in the same row indicate significant differences (*p* < 0.05).

**Table 2:** Composition and physical parameters of the PVA solutions.

Material	PVA	PVA + BT_NP_	PVA + BT_Aq_ + BT_NP_	PVA + BT_Aq_ + CA + BT_NP_
Water (% *w*/*w*)	88	68	—	—
BT_Aq_ (% *w*/*w*)	—	—	68	68
BT (% *w*/*w*)	—	20	20	20
Conductivity (μS/cm) (±1)	543 ^a^	1149 ^b^	5925 ^c^	3570 ^d^
Viscosity (cP) (±5)	283 ^a^	457 ^b^	451 ^b^	469 ^c^
pH (±0.1)	5.3 ^a^	5.2 ^a^	5.2 ^a^	3.9 ^b^

^a,b,c,d^ Different letters in the same row indicate significant differences (*p* < 0.05).

**Table 3:** Total extracted polyphenols content (TEPC) and the antioxidant activity of the obtained mats at t = 0 and aged 4 months (t = 4 months).

Material	PVA + BT_NP_	PVA + BT_Aq_ + BT_NP_	PVA + BT_Aq_ + CA + BT_NP_
TEPC (mgGAE/g of mat), t = 0	23.9 ± 0.3 ^a^	41.4 ± 0.9 ^b^	14.8 ± 0.3 ^c^
TEPC (mgGAE/g of mat), t = 4 months	23.0 ± 0.4	39.6 ± 0.5	—
Polyphenols’ retention (%)	88.9 ± 0.4 ^a^	88.2 ± 0.3 ^a^	31.0 ± 0.4 ^b^
Antioxidant activity (μmol/g of mat), t = 0	125 ± 1 ^a^	155 ± 3 ^b^	51 ± 2 ^c^
Antioxidant activity (μmol/g of mat), t = 4 months	120 ± 3 ^a^	147 ± 4 ^b^	—

^a,b,c^ Different letters in the same row indicate significant differences (*p* < 0.05).

**Table 4:** Release of polyphenols based on the TPL and the TEPC.

Films		Hydrophilic	Lipophilic	Acidic
PVA + BT_NP_	*M_∞_*/TPL (%)	67.40 ± 0.02 ^a^	80.20 ± 0.01 ^b^	54.50 ± 0.01 ^c^
	*M_∞_*/TEPC (%)	80.4 ± 0.9 ^a^	95.6 ± 0.9 ^b^	65.00 ± 0.02 ^c^
PVA + BT_Aq_ + BT_NP_	*M_∞_*/TPL (%)	76.50 ± 0.01 ^a^	86.30 ± 0.01 ^b^	60.40 ± 0.01 ^c^
	*M_∞_*/TEPC (%)	87 ± 1 ^a^	97.8 ± 0.9 ^b^	68.4 ± 0.3 ^c^
PVA + BT_Aq_ + CA + BT_NP_	*M_∞_*/TPL (%)	23.60 ± 0.01 ^a^	30.50 ± 0.01 ^b^	16.20 ± 0.01 ^c^
	*M_∞_*/TEPC (%)	75 ± 2 ^a^	98.2 ± 0.4 ^b^	51.90 ± 0.07 ^c^

^a,b,c^ Different letters in the same row indicate significant differences (*p* < 0.05).

**Table 5:** Parameters obtained by fitting the released polyphenols over time in different food simulants for the Weibull, Fick and Power Law models.

Simulant	PVA + BT_NP_			PVA + BT_Aq_ + BT_NP_			PVA + BT_Aq_ + CA + BT_NP_		
	Weibull model								
	a	b	R^2^	a	B	R^2^	a	b	R^2^
Hydrophilic	−0.49 ± 0.02	0.17 ± 0.01	0.88	−0.165 ± 0.002	0.390 ± 0.002	0.91	−0.62 ± 0.04	0.21 ± 0.01	0.79
Lipophilic	−0.350 ± 0.003	0.280 ± 0.004	0.99	−0.34 ± 0.01	0.284 ± 0.007	0.99	−0.24 ± 0.07	0.35 ± 0.04	0.99
Acid simulant	−0.8 ± 0.1	0.16 ± 0.02	0.92	−0.072 ± 0.003	0.518 ± 0.007	0.98	−0.30 ± 0.02	0.40 ± 0.03	0.83
	Fick’s model								
	D [cm^2^/s]		R^2^	D [cm^2^/s]		R^2^	D [cm^2^/s]		R^2^
Hydrophilic	1.0 ± 0.7 × 10^−15^		0.93	0.57 ± 0.01 × 10^−15^		0.88	1.7 ± 0.3 × 10^−15^		0.78
Lipophilic	0.8 ± 1.2 × 10^−15^		0.90	0.66 ± 0.04 × 10^−15^		0.80	2.4 ± 0.7 × 10^−15^		0.80
Acid simulant	1.0 ± 0.7 × 10^−15^		0.95	0.33 ± 0.09 × 10^−15^		0.70	3.3 ± 0.7 × 10^−15^		0.88
	Power Law model								
	k	n	R^2^	k	N	R^2^	k	n	R^2^
Acid simulant	0.5 ± 1.0	7484 ± 1 × 10^−5^	0.98	0.5 ± 1.0	9084 ± 7 × 10^−5^	0.94	0.6 ± 1.0	5475 ± 7 × 10^−5^	0.86

## Data Availability

Not applicable.
